# Oxidative Stress and Postoperative Outcomes: An Umbrella Review of Systematic Reviews and Meta-Analyses

**DOI:** 10.3390/antiox14111349

**Published:** 2025-11-11

**Authors:** Miguel Angel Cuevas-Budhart, María Sánchez-Garre, Alba Sánchez-Bermúdez, Aurora Sobrino-Rodríguez, María Mastel Arniella-Blanco, Alina Renghea, Almudena Crespo-Cañizares, Iván Cavero-Redondo, Juan Manuel Gallardo, Mercedes Gómez del Pulgar

**Affiliations:** 1 Unidad de Investigación Médica en Enfermedades Nefrológicas CMN Siglo XXI, Instituto Mexicano del Seguro Social, Mexico City 06600, Mexico; jmgallardom@gmail.com; 2 Grupo Quirónsalud, 28010 Madrid, Spain; mariasanchezgarre@gmail.com (M.S.-G.); sanchezbermudezalba26@gmail.com (A.S.-B.); aurora.sobrino@quironsalud.es (A.S.-R.); mastelarniella@yahoo.es (M.M.A.-B.); 3 Health Sciences Faculty, Department of Nursery, University of Francisco de Vitoria, Pozuelo de Alarcón, 28223 Madrid, Spain; a.renguea@ufv.es (A.R.); a.crespo.prof@ufv.es (A.C.-C.); mgomezdelpulgar@gmail.com (M.G.d.P.); 4 CarVasCare Research Group, Facultad de Enfermería de Cuenca, Universidad de Castilla-La Mancha, 16002 Cuenca, Spain; ivan.cavero@uclm.es

**Keywords:** oxidative stress, biomarkers, postoperative outcomes, surgery, umbrella review, patient safety

## Abstract

Background: Oxidative stress (OS) is a key biological mechanism influencing surgical recovery, contributing to impaired healing, infectious complications, cardiovascular events, and mortality. This umbrella review aimed to synthesize evidence from systematic reviews and meta-analyses exclusively focused on the relationship between validated oxidative stress biomarkers and postoperative outcomes. Narrative and non-systematic reviews were excluded. Methods: A comprehensive search of PubMed, Scopus, and Web of Science was conducted on 15 March 2024 and updated on 12 December 2024 to identify systematic reviews and meta-analyses including adult surgical patients, validated oxidative stress biomarkers, and clinical outcomes. Methodological quality was evaluated with AMSTAR 2 and ROBIS. The SANRA checklist was used only to verify that narrative or bibliometric reviews did not meet the inclusion criteria. These non-systematic reviews were excluded from the synthesis and cited solely as contextual references. Findings: From 527 records, ten systematic reviews of moderate to high methodological quality were included, encompassing approximately 230 primary studies. The most frequently reported biomarkers were total antioxidant capacity (TAC), glutathione (GSH), superoxide dismutase (SOD), malondialdehyde (MDA), and 8-hydroxy-2′-deoxyguanosine (8-OHdG). Lower TAC, GSH, and SOD levels were consistently associated with poor recovery and multiorgan dysfunction, whereas elevated MDA and 8-OHdG levels correlated with infectious complications, delayed healing, cardiovascular events, persistent pain, and mortality. Antioxidant-based interventions such as vitamin C, N-acetylcysteine, and propofol showed heterogeneous but promising effects, particularly in high-risk surgical populations. The main limitations were the heterogeneity of biomarkers, variability in perioperative protocols, and partial overlap of primary evidence across reviews. Interpretation: The findings were organized into three main clinical domains: (1) infectious complications and impaired healing; (2) cardiovascular and systemic complications; and (3) predictive and prognostic value of OS biomarkers for perioperative risk assessment. This thematic synthesis integrates evidence across different surgical specialties, highlighting consistent mechanistic patterns and key research gaps to inform future investigations and clinical decision-making.

## 1. Introduction

Oxidative stress (OS) has emerged in the last decade as a central pathophysiological factor in surgery, linked to the development of multiple postoperative complications. OS is defined as an imbalance between the excessive production of reactive oxygen species (ROS) and the endogenous antioxidant capacity to neutralize them. During a surgical procedure, ischemia, reperfusion, the systemic inflammatory response, and tissue trauma induce the overproduction of ROS, which affects lipids, proteins, and nucleic acids, with direct effects on cellular function and organic homeostasis. This state contributes to endothelial dysfunction, microcirculatory alteration, and delayed tissue repair, which increases the risk of adverse outcomes after surgery [1,2].

The observation of this phenomenon has been supported by widely validated biomarkers. Total antioxidant capacity (TAC) offers an integrated measure of the antioxidant reserve; a decrease in reduced glutathione (GSH) reflects an overload of the defensive system, as does a reduction in the activity or expression of antioxidant enzymes such as superoxide dismutase (SOD) or glutathione peroxidase (GPx) [3]. On the other hand, malondialdehyde (MDA) reflects lipid peroxidation, and 8-hydroxy-2′-deoxyguanosine (8-OHdG) indicates oxidative damage to DNA. Critical elevations or decreases in these markers have been associated with surgical site infections, multiple organ failure, cardiovascular complications, chronic postoperative pain, and oncological contexts [3,4,5].

In this regard, surgery represents a paradigmatic model of oxidative aggression. Major procedures, such as cardiac, hepatic, or oncological surgery, generate the release of free radicals, which is favored by periods of hypoxia, blood transfusions, the use of anesthetic drugs, and the sudden reperfusion of previously ischemic tissues. This oxidative cascade activates signaling pathways such as NF-κB and MAPK, which promote the release of proinflammatory cytokines, perpetuating tissue damage and potentiating postsurgical immunosuppression. Such mechanisms not only worsen the risk of immediate complications but can also condition long-term outcomes, including a greater susceptibility to infections and alterations in the evolution of chronic processes [6,7].

However, the available evidence is heterogeneous. Only systematic reviews and meta-analyses were included. Narrative and non-systematic reviews were excluded from the synthesis and were used only as contextual background, after verification with SANRA. An umbrella review was necessary because the existing evidence on oxidative stress and postoperative outcomes is fragmented across multiple systematic reviews that differ in scope, methodology, biomarkers evaluated, and surgical contexts.

This fragmentation limits the ability to establish solid clinical conclusions and to design evidence-based preventive interventions. In this scenario, umbrella reviews represent a valuable methodological tool, as they enable the critical and hierarchical synthesis of systematic reviews, integrating findings and providing a global perspective on the relationship between OS and postoperative outcomes [8,9].

The objective of this review was to integrate the available evidence from systematic reviews on the association between OS and postoperative complications. This was achieved by identifying the most studied biomarkers, relevant clinical outcomes, and the methodological quality of the studies, to inform future perioperative management strategies that enhance the safety of surgical patients.

## 2. Materials and Methods

### 2.1. Methods Design

This umbrella review was designed and conducted in accordance with the PRISMA 2020 recommendations [10] for systematic reviews and meta-analyses, as well as the JBI Manual for Umbrella Reviews, which was retrospectively registered as part of an institutional protocol [11,12].

### 2.2. Selection Criteria

Only systematic reviews, with or without meta-analysis, were considered eligible if they included adult surgical populations (≥18 years), evaluated validated oxidative-stress biomarkers such as TAC, GSH, SOD, MDA, or 8-OHdG, and reported postoperative clinical outcomes. Studies were excluded if they focused exclusively on non-surgical populations, did not evaluate oxidative-stress biomarkers, restricted outcomes to molecular or laboratory markers without clinical correlation, or were narrative and bibliometric reviews with insufficient methodological rigor. The latter were considered solely as a contextual and complementary contribution, intended to illustrate the evolution of research in the field and highlight general trends, but without directly contributing to the evidence synthesis or quality assessment.

To avoid scope drift and ensure comparability across reviews, the included systematic reviews were classified into three predefined categories according to their primary context and outcomes of interest: (i) surgical and biomarker-focused reviews, (ii) ICU/sepsis/inflammation-focused reviews, and (iii) oxygen- or intervention-focused reviews. Classification was based on the reported study populations, clinical settings, and types of outcomes evaluated. A summary table and a Venn diagram were created to visualize the distribution of reviews across these categories.

### 2.3. Information Sources and Search Strategy

A systematic search of PubMed, Scopus, and Web of Science was performed on 15 March 2024 and updated on 12 December 2024 to include the most recent literature. Complete search strategies, including keywords, Boolean operators, and filters, are listed in Supplementary Table S1. The high number of duplicate records is due to overlapping indexing across these databases and the two different search periods. Duplicates were removed following PRISMA 2020 guidelines to ensure traceability and transparency.

### 2.4. Study Selection

Titles, abstracts, and full texts were evaluated by two independent reviewers. Discrepancies were resolved through consensus with a third reviewer. Mendeley was used for reference management and decision-making records.

### 2.5. Quality and Risk of Bias Assessment

Two reviewers independently assessed methodological quality and risk of bias using the AMSTAR 2 tool (for systematic reviews) [13], ROBIS (for risk of bias in reviews) [14], and SANRA (for narrative reviews) [15]. Discrepancies were resolved through discussion or consultation with a third reviewer. To evaluate inter-rater reliability, Cohen’s κ was calculated for each tool across domains. Reviews rated as low or critically low quality were excluded from the final synthesis, ensuring that only evidence of moderate or high quality contributed to the conclusions.

### 2.6. Results Synthesis

Data extraction included key variables such as author, year of publication, country, number of primary studies, type of surgery, biomarkers evaluated, reported complications, main findings, and methodological quality. The robustness of this umbrella review was ensured through an explicit protocol, independent double evaluation, the use of validated tools, and complete adherence to international reporting standards.

Due to the heterogeneity observed in the surgical contexts, biomarker measurement methods, and clinical outcomes analyzed, a structured narrative synthesis was conducted. The findings were organized into three main clinical areas: (1) infectious complications and healing alterations; (2) cardiovascular and systemic complications; and (3) the predictive value of biomarkers and risk assessment strategies. This thematic approach enabled the integration of findings across different surgical specialties, facilitating the identification of consistent patterns and essential knowledge gaps to guide future lines of research and clinical practice.

### 2.7. Assessment of Study Overlap (CCA)

To assess the potential redundancy between systematic reviews, we constructed an overlap matrix including all primary studies (identified by first author and year) across the eligible reviews. Based on this matrix, we calculated the Corrected Covered Area (CCA), a standard metric to quantify the degree of overlap, and the Repeated Number of Overlap (RNO). In addition, we generated a network plot using networkx in Python 3.13.7., where each node represents a review and edges indicate the number of primary studies shared between pairs of reviews. Node size was proportional to the number of included studies, and edge thickness reflected the number of overlapping studies.

### 2.8. Construction of the Evidence Map

To enhance the interpretability of the findings, we constructed an evidence map in the form of a bubble plot. Each included review was classified according to its clinical context or surgical specialty (x-axis) and the main clinical outcome assessed (y-axis). Bubble size was proportional to the number of primary studies included in the review, while bubble color reflected the overall methodological quality, combining AMSTAR 2 and ROBIS assessments into three levels of concern: low (green), moderate (yellow), or high (red).

### 2.9. Certainty of Evidence and Credibility Grading

Certainty of evidence was appraised using a GRADE framework adapted for umbrella reviews. We integrated review-level quality (AMSTAR 2, ROBIS), inconsistency (direction and effect size), indirectness (population, biomarker specificity), imprecision (sample size, confidence intervals, trial sequential analysis when available), and publication bias. Overlap sensitivity was incorporated using the CCA to minimize double-counting. Evidence was classified as high, moderate, low, or very low.

## 3. Results

The study selection process was conducted in accordance with the guidelines of the PRISMA 2020 statement to ensure transparency and traceability at each stage of the analysis. A total of 527 references were identified through systematic searches in PubMed, Scopus, and Web of Science, along with manual records after removing 110 duplicates; 417 unique references were evaluated.

**Figure 1:**
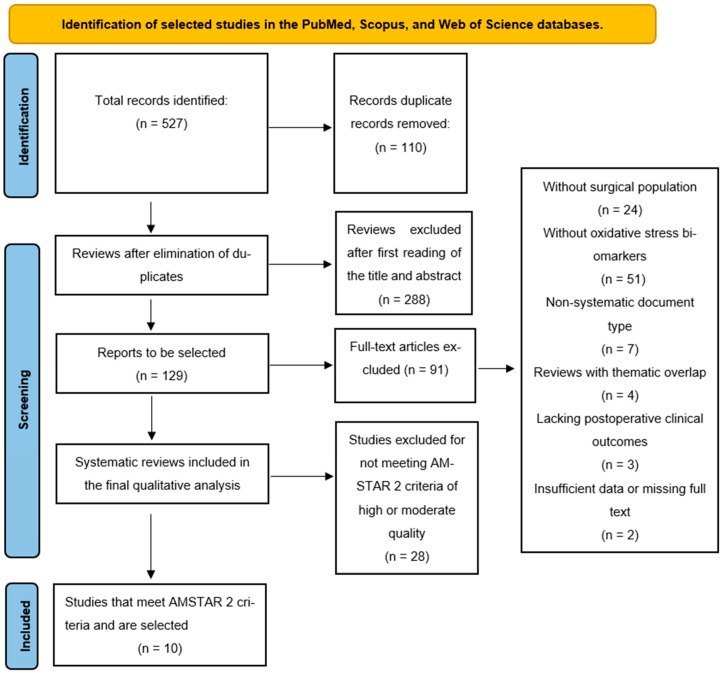
PRISMA flow diagram. Source: Prepared by the authors: Source: Page MJ, et al. BMJ 2021;372:n71. doi: 10.1136/bmj.n71 [10].

In the first screening phase, 288 articles were excluded based on title and abstract because they did not address oxidative stress biomarkers or meet the population or objectives of this review, leaving 129 studies for full-text reading. In this process, 91 articles were discarded due to the absence of biomarkers, lack of clinical outcomes, a non-surgical population, or methodological deficiencies. With the AMSTAR 2 tool, only high- or moderate-quality reviews were selected, resulting in the final inclusion of 10 high-quality systematic and narrative reviews, which form the main body of analysis. The flow diagram is shown in Figure 1 (PRISMA).

Table 1a,b summarize the methodological characteristics of the studies published between 2010 and 2025, including publication year, country, authors, study design, main findings, methodological quality according to AMSTAR 2, and risk of bias assessed with the ROBIS tool. For clarity, the studies are presented in two groups: Table 1a (systematic reviews and meta-analyses) and Table 1b (systematic and narrative reviews).

### 3.1. Methodological Quality and Risk of Bias

Domain-level results for AMSTAR 2, ROBIS, and SANRA are presented in Supplementary Table S2. Inter-rater agreement was substantial to almost perfect across most domains, with Cohen’s κ values ranging from 0.72 to 0.86.

### 3.2. Conceptual Classification of Included Reviews

The ten reviews included in our analysis were grouped into three distinct categories: four focused on surgical populations and oxidative stress biomarkers [16,17,23,25]; two centered on ICU or sepsis populations [18,20]; and four addressed oxygen-fraction interventions or general antioxidant strategies [19,21,22,24].

**Figure 2:**
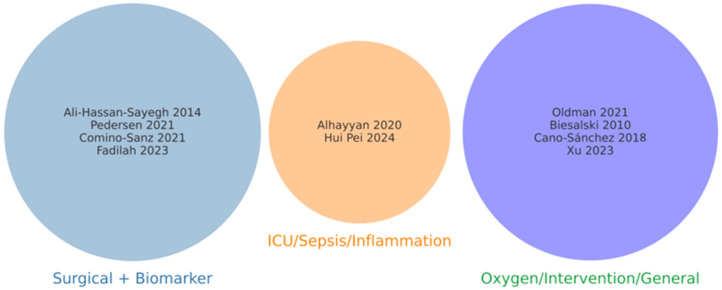
Conceptual classification of the included systematic reviews. Venn-type diagram illustrating the distribution of the ten included reviews into three predefined evidence categories: Surgical + Biomarker (blue), ICU/Sepsis/Inflammation (orange), and Oxygen/Intervention/General (purple). The classification highlights the distinct clinical contexts addressed by each group, minimizing scope drift and improving interpretability across evidence domains. Ali-Hassan-Sayegh et al., 2014 [16]; Pedersen et al., 2021 [17]; Comino-Sanz et al., 2021 [23]; Fadilah et al., 2023 [25]; Alhayyan et al., 2020 [18]; Hui Pei et al., 2024 [20]; Oldman et al., 2021 [19]; Biesalski et al., 2010 [21]; Cano-Sánchez et al., 2018 [22]; Xu et al., 2025 [24].

Figure 2 (Venn diagram) illustrates the conceptual classification of the ten included reviews, showing their non-overlapping distribution across three evidence domains. Although all reviews addressed oxidative stress or antioxidant-related outcomes, their clinical scope varied substantially, encompassing surgical, critical care, and general intervention contexts. This differentiation was considered during the interpretation and synthesis of findings. The complete classification and review distribution underlying this diagram are detailed in Supplementary Table S3.

### 3.3. Study Overlap and Redundancy

The studies encompass various surgical specialties: gastrointestinal, cardiac, vascular, orthopedic, and general surgery procedures. This variety provides a comprehensive overview of how oxidative stress impacts surgical practice, while also highlighting the heterogeneity that should be considered when interpreting the findings.

In the Asian context, several recent reviews underscore the growing prominence of the region, particularly China, in research on oxidative stress and surgical complications. Xu et al. [24] published a systematic narrative review focused on vascular diseases, in which a beneficial effect of antioxidants on oxidative stress is documented, although with still limited clinical evidence. In contrast, Pei et al. [20], through a systematic review and meta-analysis of 60 studies including 130,986 patients, reported a significant reduction in hospital and 28-day mortality, with a particularly notable benefit for ascorbic acid in subgroup analyses. Additionally, Alhayyan et al. [18] analyzed 27 randomized controlled trials and demonstrated that propofol consistently reduces postoperative oxidative stress compared with inhalational anesthesia, decreasing MDA and IL-6 while increasing SOD.

Finally, Fadilah et al. [25], from Malaysia and India, conducted a critical review of antioxidant biomaterials in tissue healing and regeneration, recognizing their therapeutic potential while also acknowledging the limited clinical evidence available. Overall, these contributions highlight the significant research activity in Asia and consolidate its strategic role in generating evidence on oxidative stress and postoperative outcomes.

**Figure 3:**
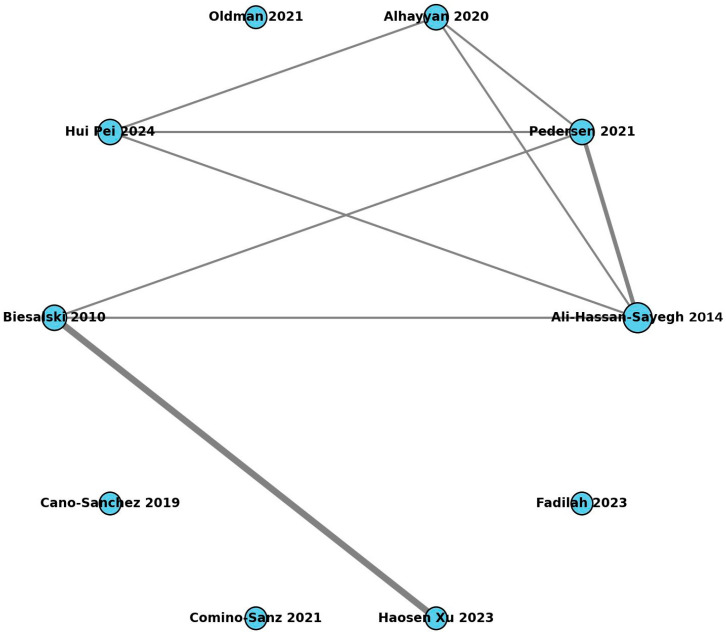
Network of overlapping systematic reviews included in the umbrella analysis. Network plot showing overlap among ten systematic reviews. Each node represents a review, and edges indicate shared primary studies; edge thickness denotes the number of duplicate citations. A total of *R* = 10 reviews included *n* = 39 unique studies and *O* = 47 appearances (*CCA* = 2.28%), indicating minimal redundancy. Reviews included in the network: Ali-Hassan-Sayegh et al., 2014 [16]; Pedersen et al., 2021 [17]; Alhayyan et al., 2020 [18]; Oldman et al., 2021 [19]; Pei et al., 2024 [20]; Biesalski et al., 2010 [21]; Cano-Sánchez et al., 2018 [22]; Comino-Sanz et al., 2021 [23]; Xu et al., 2025 [24]; Fadilah et al., 2023 [25].

The overlap matrix comprised N unique primary studies distributed across R reviews, with a total of *O* occurrences. The overall CCA was 2.28%, indicating a low degree of redundancy. The network plot (Figure 3) illustrates the relationships between reviews, showing that most shared few or no primary studies, except for a small cluster with moderate overlap. The complete citation matrix used to calculate the CCA is provided in Supplementary Table S4.

### 3.4. Evidence Mapping Results

**Figure 4:**
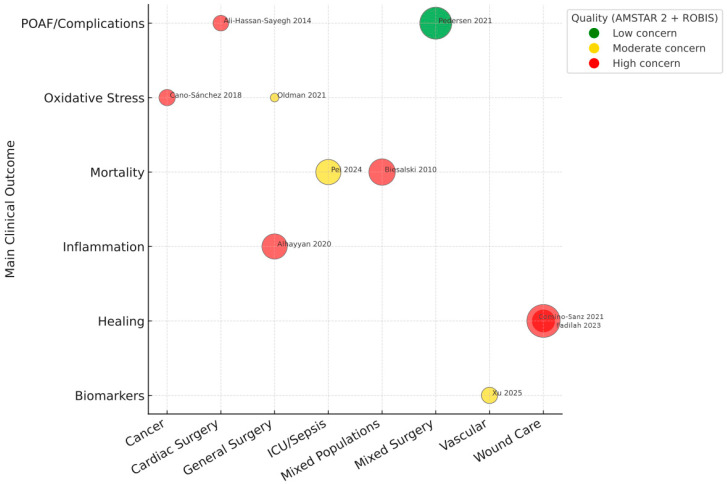
Evidence map of the included systematic reviews. Bubble plot showing the clinical focus (x-axis) and main outcomes (y-axis) of the ten included systematic reviews. Bubble size indicates the number of primary studies, and color reflects methodological quality (AMSTAR 2 + ROBIS): green = low, yellow = moderate, and red = high concern. Reviews included in the evidence map: Ali-Hassan-Sayegh et al., 2014 [16]; Pedersen et al., 2021 [17]; Alhayyan et al., 2020 [18]; Oldman et al., 2021 [19]; Pei et al., 2024 [20]; Biesalski et al., 2010 [21]; Cano-Sánchez et al., 2018 [22]; Comino-Sanz et al., 2021 [23]; Xu et al., 2025 [24]; Fadilah et al., 2023 [25].

Figure 4 displays the evidence map of the ten included reviews. Reviews were distributed across a range of contexts, from cardiac surgery and perioperative care to intensive care/sepsis and wound healing. The largest bubbles correspond to reviews with the highest number of included studies [17,20,25], whereas smaller bubbles reflect more focused syntheses [19]. Bubble colors highlight that only one review [17] was rated as low concern across both AMSTAR 2 and ROBIS, while most reviews were classified as high concern, with three placed in the moderate concern category.

### 3.5. Biomarkers and Clinical Outcomes

Table 2 summarizes the main oxidative stress biomarkers reported in the included reviews, the surgical specialties in which they were evaluated, and the associated postoperative complications. The most frequently analyzed markers were SOD and MDA, often linked to increased oxidative damage and impaired recovery. Other relevant parameters included carbonyls, NO, NF-κB, MAPK, Nrf2-ARE, NLRP3, IL-6, CRP, and WBC, which reflect inflammatory activation and redox imbalance. Several reviews also emphasized clinical outcomes such as mortality, POAF, myocardial infarction, stroke, and serious adverse events as indirect correlates of oxidative stress.

Supplementary Table S5 summarizes the methodological aspects of biomarker assessment, covering assay techniques, biological matrices, sampling times, and measurement units. Serum and plasma were the most frequent matrices, though some studies used tissue, urine, or exhaled breath condensate. Methods ranged from spectrophotometry and ELISA to HPLC, immunohistochemistry, or electrochemical assays. Sampling times varied across preoperative, intraoperative, and postoperative phases, while measurement units ranged from absolute concentrations (µmol/L, ng/mL) to relative activity indices. This heterogeneity limits comparability across reviews and highlights the need for standardized protocols in biomarker evaluation.

MDA was consistently associated with increased oxidative stress and a higher incidence of surgical infections, delayed healing, and cardiovascular complications [19]. In contrast, TAC, GSH, and SOD were associated with a protective antioxidant profile, so their decrease was connected to organ dysfunction and poor recovery [17,23]. 8-OHdG showed a close association with chronic oxidative damage and persistent pain, especially in orthopedic contexts [9,26]. Finally, IL-6 and CRP emphasized the interaction between inflammation and oxidative stress, establishing themselves as predictors of systemic complications [2,18].

### 3.6. Graded Certainty by Outcome, Specialty, and Biomarker

Table 3 shows the graded certainty of evidence across outcomes, specialties, and biomarkers. Evidence grading indicated that malondialdehyde (MDA) was consistently associated with higher infectious complications (moderate certainty). Decreased TAC, GSH, and SOD were linked to organ dysfunction and poor recovery (moderate certainty, downgraded for assay and timing heterogeneity). Associations for 8-OHdG with persistent pain were inconsistent (low certainty). In cardiac surgery, antioxidant strategies (vitamin C, NAC, PUFA) showed a reduction in POAF (moderate certainty), though small-study effects were noted. Mortality outcomes remained of low-to-moderate certainty across specialties.

Summary of GRADE assessments adapted for umbrella reviews. Certainty ratings (high, moderate, low, very low) were determined considering methodological quality (AMSTAR 2, ROBIS), inconsistency, indirectness, imprecision, publication bias, and overlap sensitivity (CCA). MDA was consistently associated with infectious complications (moderate certainty), TAC/GSH/SOD with organ dysfunction (moderate certainty), 8-OHdG with persistent pain (low certainty), antioxidant strategies with POAF reduction in cardiac surgery (moderate certainty), and mortality outcomes with low-to-moderate certainty across specialties.

### 3.7. Principal Findings by Thematic Areas

The results were grouped into three main clinical domains:

#### 3.7.1. Infectious Complications and Delayed Healing

Excessive ROS (reactive oxygen species) are reported to disrupt angiogenesis, collagen remodeling, and tissue regeneration, which favors chronic inflammation and hinders wound closure. Topical treatments such as curcumin, N-acetylcysteine, and quercetin, administered via hydrogels or nanomaterials, have shown promising preclinical results in modulating the oxidative microenvironment [20,23,25].

#### 3.7.2. Cardiovascular and Systemic Complications

Regarding cardiovascular complications, it has been documented that supplementation with vitamin C, N-acetylcysteine, and polyunsaturated fatty acids reduces mortality and the incidence of postoperative atrial fibrillation, as well as shortens hospital stays in cardiac surgery [16,17]. On the other hand, it was confirmed that ascorbic acid significantly decreased hospital and 28-day mortality, reinforcing its therapeutic potential in settings of high clinical vulnerability [20,24].

#### 3.7.3. Risk Prediction and Personalized Perioperative Management

The use of propofol is linked to lower levels of IL-6 and CRP compared to inhalational anesthesia, suggesting a role for biomarkers in the selection of anesthetic strategies [18]. Furthermore, the redox profile can assist in preoperative risk assessment and help select antioxidant therapies for high-risk surgical patients [22].

#### 3.7.4. Methodological Assessment

Five of the eleven included reviews were classified as having high methodological quality, primarily those that conducted structured meta-analyses [16,17,18,19,20]. The remaining six were considered to be of moderate quality due to limitations such as the absence of a quantitative analysis, a lack of protocol registration, or an incomplete bias assessment [21,22,23,24,25]. According to the ROBIS tool, four reviews presented a low risk of bias [17,18,20,23], while the rest showed a moderate risk [16,19,21,24,25].

## 4. Discussion

The synthesis of eleven high- and moderate-quality reviews suggests that oxidative stress is a significant pathophysiological mechanism influencing postoperative outcomes across various surgical specialties. However, the strength of this association depends on the methodological quality and risk of bias of the included reviews. By integrating evidence from systematic reviews and meta-analyses of moderate to high quality, we found consistent associations between decreased antioxidant biomarkers (TAC, GSH, and SOD) and poor recovery, and between elevated oxidative damage markers (MDA and 8-OHdG) and adverse outcomes such as delayed healing, infectious complications, cardiovascular events, persistent pain, and mortality. This conclusion is strengthened by its repeated observation across diverse settings, from cardiac surgery to care for critically ill patients, which supports the external validity of these findings.

In line with previous systematic reviews, our findings confirm that oxidative stress plays a decisive role in postoperative outcomes across surgical settings. Evidence from large reviews supports these patterns: antioxidant supplementation with vitamin C or N-acetylcysteine has been associated with reduced mortality and postoperative atrial fibrillation [17], and propofol anesthesia has shown lower oxidative and inflammatory responses compared with inhalational agents [18]. Similar benefits of antioxidant strategies have been reported in septic or critically ill patients [20], and in the context of wound healing, the use of antioxidant biomaterials has demonstrated potential to accelerate recovery [23,25]. These converging results reinforce the clinical value of addressing oxidative stress as a modifiable factor in perioperative management and patient safety.

Various reviews have underlined the importance of oxidative stress in healing processes and in susceptibility to infections. It has been demonstrated that the accumulation of reactive oxygen species interferes with angiogenesis and delays wound closure [23], a finding supported by evidence on antioxidant biomaterials, which show significant therapeutic potential for enhancing tissue regeneration [25]. Collectively, these findings suggest that controlling oxidative stress not only has preventive value but could also be incorporated as part of therapeutic management in complex surgical wounds.

Regarding systemic complications, significant reductions in mortality were documented by Pedersen et al. [17] in patients treated with vitamin C and N-acetylcysteine. However, it was recognized that this effect is attenuated when only low-risk-of-bias studies are included. Evidence in favor of antioxidants in critically ill patients with sepsis was also provided by Pei et al. [20], reported a significant reduction in hospital and 28-day mortality in critically ill septic patients receiving antioxidant supplementation, reinforcing the therapeutic potential of ascorbic acid in vulnerable populations. In contrast, consistent clinical benefits were not identified by other analyses, which underscores the methodological heterogeneity and the need for more homogeneous trials.

Additionally, in the anesthetic setting, other examples are provided of how redox control can be integrated into clinical practice. Alhayyan et al. [18] demonstrated that intravenous anesthesia with propofol is associated with lower concentrations of interleukin-6 and C-reactive protein compared to inhalational anesthesia, indicating that the choice of anesthetic agent directly influences the magnitude of the postoperative inflammatory response. Additionally, Oldman et al. [19] issued a warning regarding the risk of intraoperative hyperoxia, with high concentrations of inspired oxygen being linked to an increased production of malondialdehyde and carbonyls. These findings suggest the need for a revision of routine ventilatory strategies.

Discrepancies that limit the generalizability of the results are also revealed by the reviews. Cano-Sánchez et al. [22] emphasized that, although efficacy has been demonstrated by certain mitochondrial antioxidants in animal models, their translation to clinical practice remains limited. Biesalski et al. [21], for their part, concluded that the benefits of antioxidant supplementation are concentrated in malnourished patients or those with high basal oxidative stress, thereby restricting the universal applicability of these interventions. These divergences illustrate the complexity of translating evidence into surgical practice and underscore the need to identify which patients truly benefit from these strategies.

It is confirmed by the methodological analysis that, although several reviews achieved a high quality according to AMSTAR 2 [13], the majority showed deficiencies, such as an absence of protocol registration, scarce evaluation of publication bias, and significant heterogeneity in the biomarkers used. Another source of uncertainty is added by the overlap of primary studies, which was evaluated using the corrected covered area index, as specific findings may be overrepresented. These methodological limitations require that the results be interpreted with caution and that reviews with greater robustness be prioritized.

### 4.1. Clinical Implications

The evidence synthesized in this umbrella review reveals that oxidative stress constitutes not only a relevant pathophysiological mechanism in the perioperative period but also an element with clear implications for clinical practice. The integration of oxidative stress into the assessment and management of surgical patients could improve the precision of risk stratification, optimize therapeutic decision-making, and promote postoperative recovery, thus reinforcing patient safety. In this context, a more precise risk stratification and the guidance of personalized interventions could be permitted by the incorporation of oxidative stress biomarkers into the preoperative assessment.

(1) Incorporation of Biomarkers in Preoperative Assessment
  ○ The measurement of TAC, GSH, SOD, MDA, and 8-OHdG constitutes a valuable tool for identifying patients with a higher probability of complications, allowing for a more preventive and individualized approach based on risk.
(2) Personalized Antioxidant Interventions
  ○ The use of vitamin C and N-acetylcysteine is supported by evidence in critical or septic patients. At the same time, intravenous anesthesia with propofol offers advantages over inhalational anesthesia in surgeries with a high inflammatory risk. Likewise, the careful titration of intraoperative oxygen therapy is essential to avoid hyperoxia and reduce lipid peroxidation.
(3) Innovation in Antioxidant Biomaterials and Dressings
  ○ New perspectives are opened in reconstructive surgery and in the management of complex wounds by the development of hydrogels and dressings enriched with antioxidants, particularly in patients with comorbidities such as diabetes or malnutrition.
(4) Multidisciplinary Approach
  ○ The successful implementation of these strategies relies on coordinated participation from surgeons, anesthesiologists, intensivists, and nursing professionals, guaranteeing an integral management of oxidative stress and favoring safer surgical outcomes.

### 4.2. Recommendations for Future Research

Priority areas for scientific development regarding oxidative stress in the surgical field are identified by the findings of this review. The standardization of measurement techniques and the establishment of clinically significant reference values for biomarkers such as TAC, GSH, SOD, MDA, and 8-OHdG are fundamental, which would facilitate the comparability of studies and their application in clinical practice. Multicenter clinical trials with registered protocols, adequate sample sizes, and analyses stratified by specialty are also required to allow for the precise determination of the efficacy and safety of antioxidant interventions. Likewise, the incorporation of cost-effectiveness evaluations is necessary to assess the relevance of integrating biomarkers and antioxidant therapies into enhanced recovery after surgery (ERAS) protocols. Another pending line of research is the inclusion of vulnerable populations, such as the elderly, patients with multimorbidity, malnutrition, or emergency surgeries, who have been sparsely represented despite their greater susceptibility to oxidative damage.

The relevance of oxidative stress in postoperative complications is not only confirmed by the findings of this review, but a prospective perspective for its integration into clinical practice is also opened. The standardization of biomarkers such as TAC, GSH, SOD, MDA, and 8-OHdG will allow for the development of risk stratification algorithms and the Design of personalized antioxidant interventions. In this way, future research can consolidate prevention and management strategies that increase the safety of surgical patients and optimize postoperative recovery.

### 4.3. Study Limitations

This umbrella review presents several methodological limitations that should be acknowledged. The first limitation relates to the heterogeneity of biomarkers, surgical specialties, and clinical outcomes, which prevented direct quantitative synthesis and limited comparability across reviews. Second, the methodological quality of the included reviews varied from moderate to high according to AMSTAR 2 and ROBIS assessments, introducing variability in the strength and reliability of the evidence. Third, although the calculated CCA = 2.28% indicates a low degree of overlap, partial redundancy among primary studies may still have influenced the synthesis. Fourth, publication bias could not be excluded, since most reviews were based on published studies and English-language databases, possibly underrepresenting negative or unpublished results. Finally, as an umbrella review, the conclusions are derived from secondary sources rather than primary data, which inherently limits the depth of causal inference and the certainty of the evidence according to GRADE criteria adapted for umbrella reviews.

These limitations emphasize the need for standardized biomarker measurement protocols, prospective multicenter studies, and consistent methodological reporting to strengthen future research on oxidative stress and perioperative outcomes.

## 5. Conclusions

Overall, the evidence synthesized in this umbrella review indicates that oxidative stress is a decisive pathophysiological axis in the onset of postoperative complications and a key determinant of surgical patient safety. The findings highlight the potential of redox biomarkers, still underutilized in clinical practice, to improve risk stratification and guide personalized perioperative management. Integrating the evaluation and control of oxidative stress into surgical protocols could optimize postoperative outcomes and strengthen patient safety. Moving toward the standardization and clinical application of these biomarkers will contribute to a more precise, biologically informed, and safer model of surgical care.

The conclusions of this review should be interpreted with caution, considering the heterogeneity and methodological variability of the included evidence.

## Figures and Tables

**Table 1:** (**a**) Classification of the works under study: Systematic reviews and meta-analyses. Source: Prepared by the authors. (**b**) Classification of the works under study: Systematic and narrative reviews. Source: Prepared by the authors.

(a)						
Year	Country	Authors	Type and Design of the Study	Main Results	Methodological Quality	ROBIS
2014	International	Ali-Hassan-Sayegh et al. [16].	Meta-analysis of 23 RCTs (4278 patients)	NAC, PUFA, and vitamin C reduce POAF. PUFA and vitamin C reduce hospital stay.	High	Low
2020	Denmark	Pedersen et al. [17]	Systematic review + meta-analysis + TSA (RCTs)	26% reduction in mortality (RR 0.74), not significant in low-bias analysis	High	Low
2020	United Kingdom	Alhayyan et al. [18]	Systematic review + meta-analysis (27 RCTs)	Propofol reduces postoperative oxidative stress compared with inhalational anesthesia (↓ MDA, IL-6, ↑ SOD)	High	Low
2021	United Kingdom	Oldman et al. [19]	Systematic review of RCTs	High FiO_2_ is associated with increased MDA and carbonyls, and it decreases antioxidant levels.	Moderate–High	Moderate
2024	China	Pei et al. [20]	Systematic review + meta-analysis (60 studies, 130,986 patients)	Significant reduction in hospital and 28-day mortality (highlighted ascorbic acid)	High	Low
2010	Germany/USA	Biesalski et al. [21]	Re-evaluation of meta-analyses (66 RCTs)	36% showed benefit; only 5% showed harm. Greater benefits in malnourished populations.	Moderate–High	Moderate
**(b)**						
**Year**	**Country**	**Authors**	**Type and Design of the Study**	**Main Results**	**Methodological Quality**	**ROBIS**
2018	France/Spain	Cano Sanchez et al. [22].	Narrative systematic review	Mitochondrial antioxidants (SkQ1, elamipretide) improve wound healing in animal models.	Moderate–High	Low
2021	Spain	Comino-Sanz et al. [23].	Narrative systematic review (46 in vitro, animal, and human studies)	Curcumin, NAC, chitosan, quercetin: Improves wound healing. Limited human evidence.	Moderate	Low
2025	China	Xu et al. [24].	Narrative systematic review	Antioxidants reduce oxidative stress in vascular diseases. Limited clinical evidence.	Moderate–High	Moderate
2023	Malaysia/India	Fadilah et al. [25].	Critical review (in vitro, in vivo, clinical)	Antioxidant biomaterials improve healing; solid clinical evidence is lacking.	Moderate–High	Moderate

TSA: Trial Sequential Analysis; RCTs: Randomized Controlled Trials; NAC: N-acetylcysteine; PUFA: Polyunsaturated Fatty Acids; POAF: Postoperative Atrial Fibrillation; SkQ1: Plastoquinone conjugated with decyl; MDA: Malondialdehyde; IL-6: Interleukin-6; SOD: Superoxide Dismutase; FiO_2_: Fraction of Inspired Oxygen. NAC: N-acetylcysteine; SkQ1: Plastoquinone conjugated with decyl; ELAM: Elamipretide.

**Table 2:** Oxidative stress biomarkers, types of surgery, and associated complications reported in systematic reviews, meta-analyses, and narrative reviews.

Authors	Type of Study (Design)	Type of Surgery/Specialty	Associated Biomarkers	Associated Complications
Ali-Hassan-Sayegh et al. [16]	Meta-analysis (23 RCTs)	Cardiac surgery	NAC, PUFA, vitamin C	Reduced POAF and hospital stay
Biesalski et al. [21]	Re-evaluation of meta-analyses (66 RCTs)	Multisystem (primary/secondary prevention)	N/A (overall mortality analysis)	Mortality; contextual benefits depending on nutritional status
Pedersen et al. [17]	Systematic review + meta-analysis + TSA (RCTs)	General/Adult surgery	Non-specific (mortality, POAF, MI, stroke)	Mortality, POAF, MI, stroke, AKI, SAE
Alhayyan et al. [18]	Systematic review + meta-analysis (27 RCTs)	General surgery (multiple types)	IL-6, CRP, WBC	Inflammatory response intensity; risk of postoperative complications
Oldman et al. [19]	Systematic review (RCTs)	Non-cardiac surgery (cesarean, colorectal)	MDA, carbonyls, xanthine oxidase, SOD, TAS	Increased oxidative stress; potential adverse effects
Pei et al. [20]	Systematic review + meta-analysis (60 studies, 130,986 patients)	Sepsis (ICU, adults)	N/A (clinical variables: hospital and 28–30-day mortality)	Mortality, multiorgan dysfunction
Cano Sánchez et al. [22]	Narrative systematic review	Chronic wound healing (diabetes, hypoxia)	ROS, inflammation, NLRP3, Nrf2-ARE	Delayed healing, chronic inflammation, cellular senescence
Comino-Sanz et al. [23]	Narrative systematic review	Skin wounds (animal and human models)	SOD, lipid peroxidation, cell proliferation	Slow wound closure, low angiogenesis, inflammation
Xu et al. [24]	Systematic narrative review	Vascular surgery	ROS, NO, NF-κB, MAPK, apoptosis	Thrombosis, aneurysms, diabetic foot, vascular inflammation
Fadilah et al. [25]	Narrative and systematic review	Skin wounds and tissue regeneration	Collagen, angiogenesis, inflammation, oxidative stress	Ineffective healing, persistent inflammation

AKI: acute kidney injury; CRP: C-reactive protein; IL-6: interleukin-6; MAPK: mitogen-activated protein kinase; MDA: malondialdehyde; MI: myocardial infarction; N/A: not applicable; NF-κB: nuclear factor kappa B; NLRP3: NOD-like receptor family pyrin domain containing three inflammasome; NO: nitric oxide; Nrf2-ARE: nuclear factor erythroid 2–related factor 2/antioxidant response element; POAF: postoperative atrial fibrillation; PUFA: polyunsaturated fatty acids; RCTs: randomized controlled trials; ROS: reactive oxygen species; SAE: serious adverse events; SOD: superoxide dismutase; TAS: total antioxidant capacity; TSA: trial sequential analysis; WBC: white blood cells.

**Table 3:** Certainty of evidence across outcomes, surgical specialties, and biomarkers.

Outcome	Specialty	Biomarker (s)	No. SRs	Consistency	AMSTAR 2/ROBIS	Overlap (CCA)	Certainty (GRADE)	Notes
Infectious complications	General surgery	MDA ↑	2 SRs	Consistent ↑ risk	Moderate/Low	Low	Moderate	Heterogeneity in assays/timing
Organ dysfunction	Mixed	TAC ↓, GSH ↓, SOD ↓	3 SRs	Consistent	High/Low	Low	Moderate	Timing variability
Persistent pain	Orthopedic	8-OHdG ↑	1 SR	Inconsistent	Moderate/Moderate	Low	Low	Imprecision, indirectness
POAF	Cardiac	Vit C, NAC, PUFA	2 SRs	Moderate ↓ risk	High/Low	Low	Moderate	Small-study bias
Mortality	Sepsis/Cardiac	Multiple	3 SRs	Variable	Mixed	Low	Low–Moderate	Fragile estimates

Note: Upward arrows (↑) indicate increased biomarker levels associated with the outcome, whereas downward arrows (↓) indicate decreased levels. The notation is included to improve readability and comparison across systematic reviews.

## Data Availability

As this work is an umbrella review, all studies included in the analysis are publicly available through open-access sources.

## Supplementary Materials

The following supporting information can be downloaded at https://www.mdpi.com/article/10.3390/antiox14111349/s1. Table S1: Complete Database Search Strategies, Boolean Operators, and Applied Filters; Table S2: Domain-level ratings of methodological quality (AMSTAR 2) and risk of bias (ROBIS) across included reviews; Table S3: Classification of the included systematic reviews according to population context, main focus, and evidence category; Table S4: Citation matrix of primary studies included across four systematic reviews; Table S5: Assay Methods, Biological Matrices, Sampling Times, and Units for Each Oxidative Stress Biomarker.

## Abbreviations

8-OHdG	8-hydroxy-2′-deoxyguanosine
AKI	Acute kidney injury
AMSTAR 2	A Measurement Tool to Assess Systematic Reviews 2
CCA	Corrected Covered Area
CRP	C-reactive protein
ELAM	Elamipretide
ERAS	Enhanced Recovery After Surgery
FiO_2_	Fraction of inspired oxygen
GPx	Glutathione peroxidase
GSH	Glutathione
IL-6	Interleukin-6
MAPK	Mitogen-activated protein kinase
MDA	Malondialdehyde
MI	Myocardial infarction
NAC	N-acetylcysteine
NF-κB	Nuclear factor kappa B
NLRP3	NOD-like receptor family pyrin domain containing 3 inflammasome
NO	Nitric oxide
Nrf2-ARE	Nuclear factor erythroid 2–related factor 2/antioxidant response element
OS	Oxidative stress
POAF	Postoperative atrial fibrillation
PUFA	Polyunsaturated fatty acids
RCTs	Randomized controlled trials
ROBIS	Risk of Bias in Systematic Reviews
ROS	Reactive oxygen species
SAE	Serious adverse events
SANRA	Scale for the Assessment of Narrative Review Articles
SkQ1	Plastoquinone conjugated with decyl
SOD	Superoxide dismutase
TAC	Total antioxidant capacity
TAS	Total antioxidant status
TSA	Trial sequential analysis
WBC	White blood cells
