# Supplementary material for: Oxidative Stress and Postoperative Outcomes: An Umbrella Review of Systematic Reviews and Meta-Analyses
Source: Antioxidants (Basel). 2025 Nov 11;14(11):1349. doi: 10.3390/antiox14111349 (PMC12649464; doi:10.3390/antiox14111349)
Supplement: Supplementary file 1 [file antioxidants-14-01349-s001.zip › antioxidants-3900017-supplementary.pdf]

Supplementary Table S1. Complete Database Search Strategies, Boolean Operators, and Applied Filters

**Database**	**Search String (Boolean operators)**	**Filters**	**Date of Initial Search**	**Date of Update**
**PubMed**	(“oxidative stress”[MeSH Terms] OR “oxidative stress”[Title/Abstract] OR “redox imbalance”[Title/Abstract]) AND (“postoperative complications”[MeSH Terms] OR “postoperative outcomes”[Title/Abstract] OR “surgery”[Title/Abstract] OR “surgical patients”[Title/Abstract]) AND (“systematic review”[Publication Type] OR “meta-analysis”[Publication Type] OR “systematic review”[Title/Abstract] OR “meta-analysis”[Title/Abstract])	Humans; Adults (≥18 years); English	15 March 2024	12 December 2024
**Scopus**	TITLE-ABS-KEY(“oxidative stress” OR “redox imbalance”) AND TITLE-ABS-KEY(“postoperative complications” OR “postoperative outcomes” OR “surgery” OR “surgical patients”) AND TITLE-ABS-KEY(“systematic review” OR “meta-analysis”)	Document type: Review OR Article; Language: English; Humans; Adults (≥18 years)	15 March 2024	12 December 2024
**Web of Science (WoS)**	TS=(“oxidative stress” OR “redox imbalance”) AND TS=(“postoperative complications” OR “postoperative outcomes” OR “surgery” OR “surgical patients”) AND TS=(“systematic review” OR “meta-analysis”)	Document type: Review OR Article; Language: English; Humans; Adults (≥18 years)	15 March 2024	12 December 2024

Supplementary table 2. Domain-level ratings of methodological quality (AMSTAR 2) and risk of bias (ROBIS) across included reviews.

Review (Year)	AMSTAR 2 **Protocol registered**	AMSTAR 2 **Search strategy**	AMSTAR 2 **Study selection/ Extraction**	AMSTAR 2 **Risk of bias assessment**	AMSTAR 2 **Funding reporting**	ROBI **Eligibility criteria**	ROBIS **Identification / Selection**	ROBIS **Data synthesis**	Overall (AMSTAR 2 / **ROBIS)**	**Justification (summary)**
Ali-Hassan-Sayegh [16]	No	Partial	Partial	Partial	Not reported	High	High	High	Critically Low / High	No protocol; search limited; selective outcome reporting; high concerns.
Pedersen [17]	Yes	Yes	Yes	Yes	Reported	Low	Low	Low	Low / Low	Protocol registered; comprehensive search; robust synthesis; minor limitations.
Alhayyan [18]	Yes	Partial	Yes	Partial	Not reported	Low	High	High	Moderate / High	Registered; transparent design; incomplete synthesis;

										reporting gaps.
Oldman [19]	Yes	Yes	Yes	Yes	Reported	Low	Low	Low	Moderate / Low	Strong reporting; well-conducted; minor concerns on risk-of-bias appraisal.
Pei [20]	Yes	Yes	Yes	Partial	Partial	Low	Moderate	Moderat e	Moderate / Moderate	Clear search; recent protocol; concerns about synthesis transparency.
Biesalski [21]	No	Partial	Partial	No	Not reported	High	High	High	Critically Low / High	Narrative elements; no protocol; search and synthesis not reproducible.
Cano-Sánchez [22]	No	Partial	Partial	No	Not reported	Moderate	High	High	Critically Low / High	Narrative review style; weak methods; high

										concerns on bias and synthesis.
Comino-Sanz [23]	No	Partial	Partial	Partial	Not reported	Moderate	High	High	Low / High	Non-systematic design; partial reporting; high risk in ROBIS synthesis.
Xu [24]	No	Partial	Partial	Partial	Not reported	Moderate	Moderate	High	Low / Moderate	Mechanistic focus; weak methodology; incomplete bias handling.
Fadilah [25].	No	Partial	Partial	No	Not reported	Moderate	High	High	Critically Low / High	Critical review; lacks systematic design; major limitations in synthesis.

Each review was assessed across AMSTAR 2 and ROBIS domains. Ratings (Yes/Partial/No; Low/High/Unclear) and concise justifications are provided. Domain-level assessment ensures transparency beyond overall ratings. Source: Adapted from Ali-Hassan-Sayegh et al., 2014 [16]; Pedersen et al., 2021 [17]; Alhayyan et al., 2020 [18]; Oldman et al., 2021 [19]; Pei et al., 2024 [20]; Biesalski et al., 2010 [21]; Cano-Sánchez et al., 2018 [22]; Comino-Sanz et al., 2021 [23]; Xu et al., 2025 [24]; Fadilah et al., 2023 [25].

Supplementary table S3. Classification of the included systematic reviews according to population context, main focus, and evidence category.

Review (First author, year)	Population/Context	Main focus	Category assigned
Ali-Hassan-Sayegh [16]	Surgical (CABG)	Antioxidants, oxidative stress	Surgical + biomarker
Pedersen [17]	Surgical	Antioxidants, perioperative outcomes	Surgical + biomarker
Alhayyan [18]	Mixed (surgery + ICU)	Anesthesia, inflammatory markers	ICU/inflammation
Oldman [19]	Surgical	Oxygen fraction, oxidative stress	Oxygen/intervention
Hui Pei, [20]	ICU (sepsis)	Antioxidants, mortality	ICU/sepsis
Biesalski [21]	Mixed populations	Vitamins/carotenoids, mortality	Intervention (general)
Cano-Sánchez [22]	Cancer patients	Antioxidants, oxidative stress	Non-surgical/biomarker
Comino-Sanz [23]	Wound healing	Antioxidants, tissue repair	Surgical + biomarker
Xu [24]	Cardiovascular	Antioxidants, outcomes	Intervention/general
Fadilah [25]	Wound healing	Biomaterials, antioxidants	Surgical + biomarker

CABG = coronary artery bypass grafting; ICU = intensive care unit; SR = systematic review; RCT = randomized controlled trial.

Supplementary Table S4. Citation matrix of primary studies included across four systematic reviews.

	**Ali-Hassan-Sayegh 2014**	**Pedersen 2021**	**Alhayyan 2020**	**Oldman 2021**	**Hui Pei 2024**	**Biesalski 2010**	**Cano-Sanchez 2019**	**Comino-Sanz 2021**	**Haosen Xu 2023**	**Fadilah 2023**
Ahuja 2018	0	0	0	1	0	0	0	0	0	0
Ajmal 2019	0	0	0	0	0	0	0	1	0	0
Arstall 1995	1	0	0	0	0	0	0	0	0	0
Bairati 2005	0	0	0	0	0	0	1	0	0	0
Bjordahl 2012	1	1	0	0	0	1	0	0	0	0
Busra 2017	0	0	0	0	0	0	0	0	0	1
Calo 2005	1	0	0	0	0	0	0	0	0	0
Day 2015	0	0	1	0	0	0	0	0	0	0
Fowler 2014	0	0	0	0	1	0	0	0	0	0
Gallelli 2020	0	0	0	0	0	0	0	1	0	0
Garcia-de-la-Asuncion 2007	0	0	0	1	0	0	0	0	0	0
Helmy 2000	0	0	1	0	0	0	0	0	0	0
Hercberg 2004	0	0	0	0	0	1	0	0	1	0
Hwang 2020	0	0	0	0	1	0	0	0	0	0
Jimenez-Garcia 2018	0	0	0	0	0	0	0	1	0	0
Kazemi 2013	1	0	0	0	0	0	0	0	0	0

Khaw 2002	0	0	0	1	0	0	0	0	0	0
Khaw 2008	0	0	0	1	0	0	0	0	0	0
Kim 2011	1	0	0	0	0	0	0	0	0	0
Leong 2010	0	1	0	0	0	0	0	0	0	0
Lippman 2009	0	0	0	0	0	1	0	0	1	0
Merrell 2009	0	0	0	0	0	0	0	1	0	0
Ozaydin 2013	1	0	0	0	0	0	0	0	0	0
Ozcan 2016	0	0	1	0	0	0	0	0	0	0
Pathak 2009	0	0	0	0	0	0	1	0	0	0
Rodrigo 2013	1	1	1	0	1	0	0	0	0	0
Rosengrave 2022	0	0	0	0	1	0	0	0	0	0
Safaei 2017	0	1	0	0	0	0	0	0	0	0
Sallehuddin 2022	0	0	0	0	0	0	0	0	0	1
Salonen 2003	0	0	0	0	0	1	0	0	0	0
Schneemilch 2005	0	0	1	0	0	0	0	0	0	0
Sevransky 2021	0	0	0	0	1	0	0	0	0	0
Simone 2007	0	0	0	0	0	0	1	0	0	0
Smeets 1997	0	1	0	0	0	0	0	0	0	0
Usharani 2008	0	0	0	0	0	0	0	0	1	0

Vairamon 2009	0	0	0	0	0	0	0	0	0	1
Virtamo 1994	0	0	0	0	0	1	0	0	1	0
Weijl 2004	0	0	0	0	0	0	1	0	0	0
Zhang 2021	0	0	0	0	0	0	0	0	0	1

Binary matrix displaying the presence (1) or absence (0) of individual primary studies across Oldman 2021, Pedersen 2021, Alhayyan 2019/2020, and Ali-Hassan-Sayegh 2014. This matrix was used to calculate the corrected covered area (CCA = 2.08%), indicating minimal overlap among reviews. The greatest redundancy was observed between Pedersen 2021 and Ali-Hassan-Sayegh 2014 (≥11 shared RCTs), while no overlap was detected between Oldman 2021 or Alhayyan 2019/2020 and the antioxidant-focused reviews. Source: Adapted from Ali-Hassan-Sayegh et al., 2014 [16]; Pedersen et al., 2021 [17]; Alhayyan et al., 2020 [18]; Oldman et al., 2021 [19]; Pei et al., 2024 [20]; Biesalski et al., 2010 [21]; Cano-Sánchez et al., 2018 [22]; Comino-Sanz et al., 2021 [23]; Xu et al., 2025 [24]; Fadilah et al., 2023 [25].

Supplementary Table S5. Assay Methods, Biological Matrices, Sampling Times, and Units for Each Oxidative Stress Biomarker

**Biomarker**	**Typical matrix**	**Predominant assay**	**Common units**	**Timing windows used**	**Notes on variability**	**Reported in reviews**
MDA (TBARS)	Plasma/serum/erythrocytes	TBARS (TBA adduct)	nmol/mL (μmol/L)	T0–T4	Non-specific; diet/hemolysis sensitive	Oldman 2021; narrative wound reviews
Protein carbonyls	Serum/plasma	DNPH derivatization	nmol/mg protein	T0–T3	Oxidized protein load; assay variability	Oldman 2021
F2-isoprostanes	Plasma/urine	GC-MS or LC-MS/MS	pg/mL; ng/mmol creatinine	T0–T3	Gold-standard for lipid peroxidation	(contextual)
8-OHdG	Urine/plasma	ELISA; LC-MS/MS	ng/mL; ng/mg creatinine	T0–T3	DNA oxidation; platform-dependent	Narrative reviews
SOD activity	Erythrocytes/plasma	Enzymatic activity	U/mL (U/g Hb)	T0–T3	Matrix & temperature sensitive	Oldman 2021
GPx activity	Erythrocytes/plasma	Coupled enzymatic	U/L (U/g Hb)	T0–T3	Requires GSH/NADPH coupling	Narrative reviews
Catalase	Erythrocytes/plasma	H2O2 decomposition	U/mL	T0–T3	Influenced by hemolysis	Narrative reviews
TAS/TAC*	Serum/plasma	ABTS/FRAP colorimetry	mmol Trolox eq/L	T0–T3	Platform/calibration differences	Oldman 2021; others
AOPP	Serum	Spectrophotometric	μmol/L (chloramine-T eq)	T0–T3	Reflects protein oxidation	Narrative reviews
IMA	Serum	Albumin–cobalt binding	U/mL	T0–T2	Ischemia proxy; acute shifts	COVID/surgical contexts
Total thiols	Serum/plasma	Ellman’s reagent	μmol/L	T0–T3	Nutritional status sensitive	Contextual

XO (xanthine oxidase)	Serum	Enzymatic	U/L	T0–T2	Purine metabolism; O2− source	Oldman 2021
NOx (nitrite/nitrate)	Plasma/serum	Griess reaction	μmol/L	T0–T2	Endothelial function proxy	Narrative reviews

*We harmonize to TAS/TAC (Total Antioxidant Status/Capacity) and use a single abbreviation throughout. Source: Adapted from Oldman et al., 2021 [19]; Comino-Sanz et al., 2021 [23]; Xu et al., 2025 [24]; Fadilah et al., 2023 [25]; narrative reviews cited in main text.
